# Withdrawal Dyskinesia Associated With Aripiprazole in a Child: A Case Report

**DOI:** 10.7759/cureus.65223

**Published:** 2024-07-23

**Authors:** Haruo Nishijima, Miyuki Nishijima, Chikyo Oyama, Masahiko Tomiyama

**Affiliations:** 1 Neurology, Hirosaki University Graduate School of Medicine, Hirosaki, JPN; 2 Neurology, Hirosaki University Hospital, Hirosaki, JPN; 3 Psychotherapy, Seikyoh Sakura Hospital, Aomori, JPN; 4 Psychiatry, Seikyoh Sakura Hospital, Aomori, JPN

**Keywords:** withdrawal dyskinesia, child, antipsychotic, autism, aripiprazole

## Abstract

Atypical antipsychotics are considered to be better tolerated than typical antipsychotics; however, the risk of drug-induced movement disorders needs to be considered. Aripiprazole, a dopamine partial agonist, is one of the most frequently used atypical antipsychotics in children. In this report, we describe withdrawal dyskinesia after aripiprazole discontinuation in a child with autism spectrum disorder. The patient presented with oral dyskinesia after discontinuation of aripiprazole when he was 13 years old. Dyskinetic movements disappeared after reinitiation of aripiprazole. He developed oral dyskinesia again after a reduction of the aripiprazole dose when he was 14 years old. Dyskinesia gradually disappeared within a few months. Withdrawal dyskinesia associated with aripiprazole has been rarely reported in children. Moreover, there is no large study on the prevalence of dyskinesia associated with aripiprazole discontinuation either in adults or in children. However, relevant cases might be unreported, pretermitted, or regarded as akathisia or symptoms of attention-deficit hyperactivity disorder. The prevalence of withdrawal dyskinesia associated with aripiprazole, especially in children, may be more frequent than thought. Withdrawal dyskinesia is self-limited; however, such dyskinetic movements in children potentially result in irreversible effects that damage the quality of life. As such, physicians should be mindful when changing, reducing, or discontinuing antipsychotics in children.

## Introduction

In recent years, the use of second- or third-generation (atypical) antipsychotics has increased, compared with that of first-generation (typical) antipsychotics [1]. Atypical antipsychotics, with a lower rate of drug-induced movement disorders such as parkinsonism and dyskinesia, are better tolerated. However, the risk of such disabling side effects exists. The prevalence of tardive dyskinesia in patients taking atypical antipsychotics is approximately 20% [2]. Moreover, apart from adults, antipsychotics are widely prescribed for children and adolescents. Haloperidol, a first-generation antipsychotic, reportedly induced dyskinesia in 40 of 118 (33.9%) children with autism [3]. Conversely, it is unclear how often dyskinesia develops in children undergoing atypical antipsychotic treatment. Aripiprazole, a partial dopamine agonist [4], is one of the most frequently used atypical antipsychotics in children with autism [5]. This report describes the withdrawal dyskinesia after aripiprazole discontinuation in a child with autism spectrum disorder (ASD).

## Case presentation

A boy, aged 11 years and 11 months, diagnosed with ASD and attention-deficit hyperactivity disorder (ADHD) by his pediatrician was referred to our hospital. His irritability and roughness were causing difficulties at school. Computed tomography of the brain and blood tests did not reveal anything significant. He did not exhibit abnormal involuntary movements.

**Figure 1:**
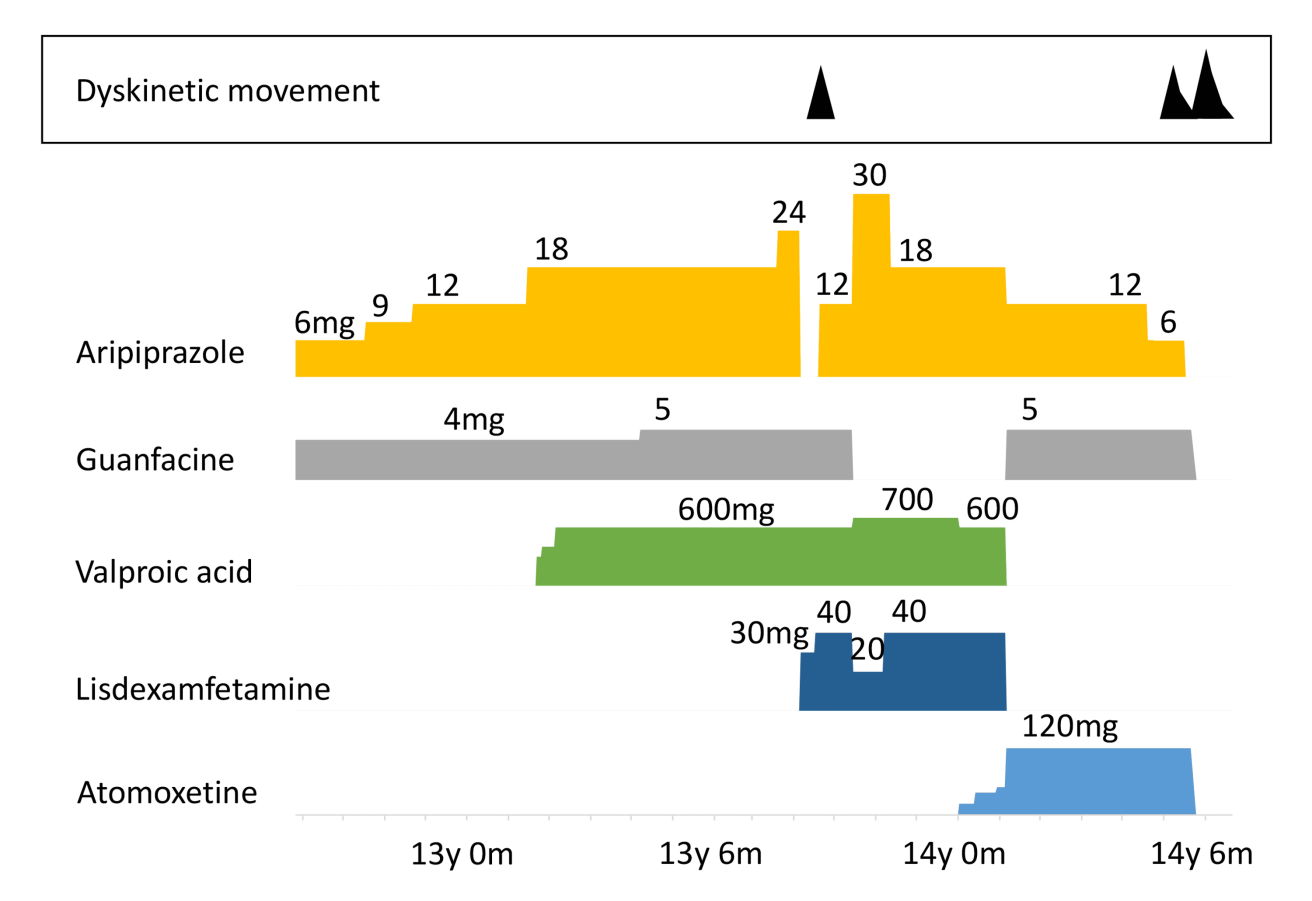
Clinical course and drug treatment during the first and second episodes of withdrawal dyskinesia associated with aripiprazole discontinuation When the patient was 13 years and eight months old, aripiprazole was discontinued and lisdexamfetamine was administered instead, then he presented with dyskinesia. Treatment with aripiprazole was resumed and the dyskinesia disappeared within two weeks. When he was 14 years and five months old, the aripiprazole dose was decreased, and then he developed dyskinesia again. When aripiprazole treatment was discontinued, the dyskinesia significantly worsened. Then atomoxetine and guanfacine were also discontinued, and the dyskinetic movement gradually disappeared within a month. y: years; m: months

At the age of 13 years and eight months, he was treated with aripiprazole (24 mg/d, in two divided doses of 12 mg), guanfacine (5 mg/d, once a day), and valproic acid (600 mg/d, in two divided doses). Aripiprazole had been gradually increased to this dose with the consent of his parents, because hyperactivity, impulsivity, hyperlogia, and stalking behavior were causing several troubles in his school life. Subsequently, aripiprazole was abruptly discontinued as it did not address his symptoms effectively, and lisdexamfetamine (30 mg/d, once a day) was administered instead. Four days after aripiprazole discontinuation, he presented with abnormal orolingual movements, that is vacuous chewing, tongue writhing, and lip licking (Figure 1). The total score of the abnormal involuntary movement scale score (AIMS) (items 1-7, score range 0-28) [6] was 5. After several days, he developed truncal choreic movement (AIMS: 8). His irritability worsened and daily activities became difficult. Therefore, treatment with aripiprazole (12 mg/d) was resumed. We chose a dose lower than the initial one because aripiprazole was re-started only as a diagnostic treatment for involuntary movements. Abnormal movements of the jaw, lip, tongue, and trunk reduced and then disappeared within two weeks (Figure 1).

At the age of 14 years and five months, he was receiving aripiprazole (12 mg/d), guanfacine (5 mg/d), and atomoxetine (120 mg/d, in two divided doses). Subsequently, the aripiprazole dose was decreased to 6 mg/d because of frequent wandering, which was considered akathisia. Seven days after the decrement, he developed orolingual dyskinesia again (Figure 1) (AIMS: 4). The dyskinesia gradually subsided within a few weeks (AIMS: 2). Subsequently, aripiprazole treatment was discontinued. Four days after discontinuation, oral dyskinetic movements worsened significantly. Apart from orolingual movements, wide opening of eyes, facial grimacing, and lower limb choreic movements also appeared on the following days (AIMS: 11). His dyskinesia improved on the day he did not take atomoxetine, whereas it worsened after taking atomoxetine. Therefore, atomoxetine and guanfacine were discontinued. The dyskinetic movement gradually disappeared within a month (AIMS: 0) (Figure 1).

The patient is currently 15 years and 10 months old, does not take any antipsychotics, and has no abnormal involuntary movements.

## Discussion

Antipsychotics-induced dyskinesia is divided into three types: tardive, covert, and withdrawal dyskinesia [7,8]. Tardive dyskinesia occurs during treatment with dopamine-blocking agents. Covert dyskinesia is disguised as dyskinesia during active antipsychotic treatment; it appears after dose reduction or discontinuation, does not remit spontaneously, and is usually permanent. Withdrawal dyskinesia initially occurs after the discontinuation of an antipsychotic agent and is self-limiting, with complete remission expected. This is considered a result of a temporary hyperdopaminergic state in the basal ganglia caused by the discontinuation of dopamine-blocking agents [7].

The majority of antipsychotics-induced dyskinesia in children involve withdrawal dyskinesia [3,9]. Only a few studies have reported dyskinesia after aripiprazole discontinuation in adults [8,10]. In children and adolescents, we could find only one case of dyskinesia associated with aripiprazole discontinuation involving a 17-year-old White male with bipolar disorder [11], which might be covert dyskinesia because the dyskinesia persisted at the last reported follow-up. The present case is perhaps the first report of the apparent withdrawal dyskinesia associated with aripiprazole discontinuation in a child. However, no large-scale studies have been conducted on the prevalence of dyskinesia associated with aripiprazole discontinuation in adults or children. Additionally, relevant cases might have been unreported, pretermitted, or regarded as akathisia or symptoms of ADHD. Thus, the prevalence of withdrawal dyskinesia associated with aripiprazole, especially in children, may be higher than previously estimated.

Our patient presented two episodes of withdrawal dyskinesia; in the first episode, aripiprazole was abruptly discontinued from a high dose, whereas in the second episode, aripiprazole was tapered. In the previous reports of dyskinesia associated with aripiprazole discontinuation, the aripiprazole dose was tapered in all the cases [8,10,11]. Moseley et al. reported an adult case with tapering of the dose from 15 mg/day over four weeks [8]. Urbano et al. reported two adult cases, in both of which the aripiprazole dose was gradually decreased from 5 mg/day to zero [10]. Even in Kafantaris et al.’s case of a 17-year-old boy, aripiprazole was tapered [11]. Thus, dyskinesia associated with aripiprazole discontinuation may occur not only with abrupt discontinuation after a high dose but also despite a cautious tapering of the dose.

The mechanisms underlying the withdrawal dyskinesia following aripiprazole discontinuation are unclear. It may be explained by the dopaminergic hypersensitivity hypothesis proposed for haloperidol-induced tardive dyskinesia [12]. In the current case, chronic administration of aripiprazole, a partial dopamine agonist, may have induced brain dopamine receptor hypersensitivity. When aripiprazole was discontinued, the antagonistic effect on dopamine receptors disappeared, and excessive dopaminergic stimulation may have induced hyperkinetic movement disorders. Moreover, drugs that enhance the dopaminergic or noradrenergic effects may enhance dyskinesia. In this case, lisdexamfetamine, atomoxetine, and guanfacine were co-administered when aripiprazole was discontinued. Lisdexamfetamine blocks dopamine and noradrenaline transporters and increases their levels in the brain [13]. Atomoxetine blocks noradrenaline transporters and increases noradrenaline levels in the brain [14]. Additionally, in the prefrontal cortex, noradrenaline transporters take up noradrenaline and dopamine [15]. In a basic animal study, atomoxetine increased dopamine levels in the prefrontal cortex [16]. Guanfacine also facilitates noradrenergic neural transmission [17]. These drugs may have worsened the dyskinesia in this case. This is supported by the fact that atomoxetine appeared to enhance dyskinetic movement in the second episode. Moreover, covert dyskinesia associated with aripiprazole discontinuation was reported in patients [8] receiving serotonin and noradrenaline reuptake inhibitors duloxetine and venlafaxine [18,19], or the dopamine and noradrenaline reuptake inhibitor methylphenidate [20]. These drugs may also have the potential to exacerbate dyskinesia.

## Conclusions

Although withdrawal dyskinesia disappears within a certain period, children and adolescents affected by it may refuse to go to school or interact with other children, risking irreversible effects. Thus, physicians must monitor for dyskinesia occurrence while changing, reducing, or discontinuing antipsychotics in children. Careful drug adjustment is imperative to avoid severe influences on patients’ lives.
